# Development and Psychometric Properties of PEACE-Q: A Questionnaire on Attitudes Towards Physician-Assisted Dying, Euthanasia, Advance Directives and Care at the End-of-Life

**DOI:** 10.7759/cureus.106265

**Published:** 2026-04-01

**Authors:** Vasilios Koulouras, Spiros Georgakis, Elena Dragioti, Foteini Veroniki, Mary Gouva

**Affiliations:** 1 Intensive Care Unit, University Hospital of Ioannina, Ioannina, GRC; 2 Faculty of Medicine, School of Health Sciences, University of Ioannina, Ioannina, GRC; 3 Research Laboratory Psychology of Patients, Families & Health Professionals, University of Ioannina, Ioannina, GRC

**Keywords:** advance directives, end-of-life care, euthanasia, icu, palliative care, physician-assisted dying

## Abstract

Public attitudes toward euthanasia, physician-assisted dying (PAD), advance directives (ADs), and end-of-life (EOL) decision-making in the ICU remain underexplored, while existing tools address these issues fragmentarily. This study aimed to develop and psychometrically evaluate a novel, self-report instrument named Physician-assisted dying, Euthanasia, Advance directives and Care at the End-of-life Questionnaire (PEACE-Q) to cohesively assess relevant public attitudes. PEACE-Q was developed through literature review, expert evaluation and pilot testing, with initial validation conducted in Greece. Psychometric evaluation included exploratory factor analysis, item response theory modeling, internal consistency testing and test-retest reliability. Data were collected from 1,046 adults residing in Greece without additional eligibility criteria. Participation was voluntary, anonymous, and based on informed consent. Analysis yielded three reliable thematic sections on public perspectives: euthanasia/PAD with three subscales, ADs with two subscales, and EOL practices and palliative care with three subscales, all demonstrating excellent internal consistency and moderate intercorrelations. Additionally, the Existential Readiness and Death Acceptance Index (ERDAI) emerged as a composite score with excellent internal consistency (α = .911), reflecting preparedness towards EOL decision-making and death acceptance. PEACE-Q proved valid, reliable and conceptually cohesive for assessing attitudes on euthanasia, PAD, ADs, and EOL practices, applicable both independently per section and comprehensively through the ERDAI, with broad applicability across diverse cultural and legal settings.

## Introduction

Recently, a marked evolution in how death is socially conceptualised and negotiated has been observed [1]. In clinical practice, despite remarkable technological advances and uprising expectations for successful outcomes across even the most invasive interventions [2], over half of critically ill patients die before hospital discharge, with nearly 20% of these deaths occurring in the intensive care unit (ICU) [3].

Hence, ICU stay remains a therapeutic trial without guarantees [4]. Aggressive interventions often do not meaningfully improve outcomes, increasing patient suffering while placing emotional and financial strain on families [5,6]. These end-of-life (EOL) situations raise complex ethical dilemmas [7]. In response, practices such as withholding or withdrawing life-sustaining treatment have been developed [8], grounded in bioethical principles and widely integrated into clinical protocols [5,9,10]. In tandem, advance directives (ADs) and decision-making models grounded on patient autonomy are also increasingly incorporated into EOL care [1,11-13].

Respect for individual autonomy also remains a key argument in favor of euthanasia and physician-assisted dying (PAD) - both of which have been linked with landmark legal cases, wide media coverage and a growing regulatory and legislative activity in some jurisdictions [14,15].

Euthanasia, PAD, and EOL decisions remain among the most ethically and socially complex areas in medicine [16,17]. Their framing and public perception vary widely worldwide, shaped by diverse cultural and systemic factors [15,17-23].

Key concepts surrounding euthanasia, physician-assisted dying and EOL decision-making remain inconsistently defined and distinguished [19,24-26], whereas public attitudes toward EOL issues are underexplored [23,27]. Literature remains fragmented, often addressing euthanasia, PAD, and EOL issues separately, with little or vague conceptual integration [25]. At the same time, existing debates mainly reflect North American or Western European settings and focus mostly on clinicians’ or relatives’ perspectives [15,16,26], limiting their relevance in culturally and systemically distinct contexts.

Across diverse national settings, euthanasia and PAD remain illegal [26], while the framework for ADs, EOL practices, and decision-making remains both underdeveloped and ambiguously defined [28-32]. Existing instruments focus on clinicians or family perspectives and do not fully address the public or all relevant concepts cohesively, even across different sociocultural and legal contexts [33-38].

Ηence, a pressing need is identified to investigate relevant public attitudes across the social spectrum in a clear, distinct and conceptually cohesive way, particularly in settings with varying regulatory frameworks and cultural backgrounds [28,29,39-42].

To address this gap, the Physician-assisted dying, Euthanasia, Advance Directives, and Care at the End-of-life Questionnaire (PEACE-Q) was developed. It is a novel self-report instrument designed with adaptability as a core feature to ensure international applicability across diverse sociocultural and legal contexts. It captures societal perspectives by addressing each thematic section autonomously, while at the same time providing a holistic existential orientation toward end-of-life issues and death, systematically integrating the ethical, legal, religious and practical dimensions that shape relevant attitudes. Its design allows adaptation to various regulatory frameworks and cultural settings, making it applicable in countries with differing approaches to euthanasia, PAD, and EOL care.

The present study pursues the following objectives: (a) to describe the development of the PEACE-Q through a systematic multistage process; (b) to evaluate its psychometric properties; and (c) to assess its validity and reliability as an instrument for measuring public attitudes toward euthanasia, PAD, ADs, and EOL practices. Initial validation was conducted within a large Greek population-based sample - a setting characterized by strong religious traditions, an underdeveloped regulatory framework and ongoing public debate on EOL issues. Beyond its research utility, findings derived from the PEACE-Q's application may inform clinical communication, ethical debate, and policymaking, and support its deployment as an international tool across diverse cultural and regulatory contexts.

## Materials and methods

Instrument development

The PEACE-Q was developed through a multistage process to ensure conceptual clarity, content validity, and psychometric robustness. Before commencing data collection, the PEACE-Q was developed following international recommendations for the design and adaptation of self-report measures [43]. The development process was structured to create a tool adaptable to various regulatory and cultural settings, while initial validation was conducted in Greece. It was constructed through a four-stage process: (a) item generation from literature review and comparative analysis of existing tools, (b) drafting of items designed for cross-cultural applicability, (c) review by an expert panel of intensivists, lawyers and psychologists, and (d) pilot cognitive debriefing with laypersons (N = 50) to ensure clarity and cultural relevance within the Greek context. The final version incorporated adjustments based on pilot feedback and was proofread for linguistic accuracy and consistency. The final design allows for adaptation to different legal frameworks - including jurisdictions where euthanasia and PAD are legal, illegal, or undergoing legislative change - while maintaining its core structure and psychometric properties. It evaluates public attitudes by addressing personal, ethical, legal and religious dimensions of the investigated concepts, aiming to explore how individuals understand, relate and respond to them within the framework of their personal values, faith, and autonomy regardless of their sociocultural background. The full English version of the instrument is available in the Appendix Section.

Ethical considerations

The study protocol was reviewed and approved by the Research Ethics Committee of the University of Ioannina (Approval No. 48956) in accordance with institutional guidelines and the principles of the 1964 Helsinki Declaration and its later amendments. Participation was strictly voluntary, anonymous, and based on informed consent.

Research context and participants

Data collection was implemented through a nationwide cross-sectional survey, disseminated across all regions of Greece. Questionnaires were distributed and collected consecutively between January and June 2025. The study population consisted of adult (≥18 years) Greek citizens, residing in Greece, regardless of sociodemographic characteristics. Only one response per individual was accepted.

The questionnaires were distributed nationwide via a secure online platform (Google Forms), accessible without login or personal identifiers to ensure anonymity. A snowball sampling strategy was applied to ensure broad participation from all geographic regions of the country. The estimated completion time was 15-20 minutes. To enable test-retest reliability analysis, participants were asked to generate a personal identification code (derived from non-identifying family initials and date of birth patterns), allowing to match responses without compromising anonymity.

Upon completion, responses were exported into Excel format, then converted into JAMOVI, SPSS, and R datasets for analysis. All data were stored on encrypted, password-protected devices accessible only to the research team.

Measurements

The introductory sociodemographic module includes items about age, sex, residence, educational status and employment. Additional items capture prior ICU exposure, professional background in healthcare, close contacts with health professionals and religiosity. All items were rated on a 5-point Likert scale and multiple-choice formats.

Section A includes 19 Likert-type items addressing conceptual distinctions between euthanasia and PAD, ethical and legal acceptability, need for legal regulation, willingness to request euthanasia/PAD in severe illness and the perceived influence of religion. This section engages with autonomy and self-determination, as well as ethical dilemmas surrounding the occurrence of death.

Section B comprised 10 Likert-type items exploring participants’ perspectives on ADs and healthcare proxies, focusing on willingness to prepare, trust in close persons as proxies, readiness to assume the role of proxy for others and the perceived need for legal regulation. This section captures both attitudes toward advance care planning and the broader ethical-legal considerations that shape decision-making at the end of life.

Section C contains 21 Likert-type items examining perceptions of treatment withdrawal, withholding of life-sustaining interventions, and Do-Not-Resuscitate orders. It assesses personal and ethical perspectives, perceived moral equivalence of different EOL practices, roles of clinicians and families in decision-making, willingness to endorse the implementation of EOL practices and palliative care, as well as attitudes toward legal regulation and religious influence.

Data analysis

An integrated analytic strategy was adopted to ensure both structural and item-level evaluation of the questionnaire. First, an exploratory factor analysis (EFA) was conducted using principal component analysis (PCA) with Varimax rotation to identify the underlying factor structure. Factors were retained based on the Kaiser criterion (eigenvalues > 1) and items were assigned to the factor on which they showed the highest loading [44,45]. This method was selected because our primary aim at this stage was exploratory - mapping the structure of attitudes rather than modelling latent constructs - and because the hypothesised sections were considered conceptually distinct. The use of PCA with orthogonal rotation facilitated a parsimonious and interpretable factor structure, supported by the large sample size. Confirmatory factor analysis (CFA) was not selected because the dimensional structure of the adapted questionnaires was not yet fully established. This choice is consistent with recommendations that EFA be used in the early stages of psychometric evaluation [44,45]. Second, Item Response Theory (IRT) was applied using the Graded Response Model (GRM), estimated separately for each subscale identified in the EFA [46,47]. Item discrimination (a) and threshold parameters (b1-b4) were estimated with an ordered logit approximation, with θ scores derived from the first principal component of each subscale. GRM provides estimates of item discrimination and thresholds, offering detailed information on how individual items perform across the latent trait continuum. Reverse-coded items were recorded to align their directionality. In addition, Cronbach’s α coefficients were computed to assess internal consistency reliability [48]. Finally, Test Information Functions (TIFs) were examined to evaluate the precision of measurement across the latent trait continuum (θ) [49]. The analyses were performed using R 4.4.1 version (lavaan, psych, mirt packages) and JAMOVI 2.6.44 version.

## Results

Participants’ characteristics

A total of N = 1046 valid responses were collected. Participants’ age ranged from 19 to 92 years (M = 46.18, SD = 13.18), with 697 (66.6%) women and 349 (33.4%) men. The majority of the participants resided in urban areas, with 912 (87.2%) living in urban settings and 134 (12.8%) in rural or semi-urban areas. Educational status was distributed as follows: 9 (0.9%) had completed primary education, 91 (8.7%) secondary education, 297 (28.4%) university or technological institute education, 406 (38.8%) postgraduate studies and 249 (23.8%) doctoral studies. Approximately 548 (52.4%) of the participants identified as healthcare professionals and 414 (39.6%) reported having a close relative previously admitted to an ICU. Religiosity was reported as high in 334 of them (31.9%), moderate in 272 (26.0%), and low or none in 440 (42.1%) of the sample.

Perspectives on euthanasia and physician-assisted dying

Exploratory Factor Analysis

Exploratory factor analysis (PCA with Varimax rotation) indicated a four-factor solution. Factor 1 included 13 items, Factor 2 comprised items A16 and A17, Factor 3 comprised A14-A15, and Factor 4 comprised A1-A2. The rotated solution explained 75.1% of total variance, revealing one dominant factor alongside narrower subdimensions (Table 1).

**Table 1:** Exploratory Factor Analysis (PCA with Varimax) – Factor Loadings Note: PCA = Principal Component Analysis. Loadings ≥ 0.30 are shown in bold. The table indicates each item's primary factor and its highest loading. *Factor 4 (items A1–A2) showed low internal consistency (α = .53) and was not reported as a separate subscale. These items are retained only in the total section score.

Item	Factor 1	Factor 2	Factor 3	Factor 4*	Primary Factor	Loading
Α2	-0.075	0.078	-0.014	-0.814	Factor4*	0.823
Α3	-0.824	-0.051	0.100	-0.073	Factor1	0.966
Α5	-0.771	-0.065	0.079	-0.057	Factor1	0.970
Α10	-0.836	-0.082	0.082	-0.064	Factor1	0.748
Α11	-0.839	-0.040	0.131	-0.032	Factor1	0.805
Α12	-0.796	-0.034	0.217	-0.013	Factor1	0.744
Α13	-0.811	-0.056	0.170	-0.028	Factor1	0.827
Α14	-0.194	-0.019	0.961	-0.010	Factor3	0.816
Α15	-0.200	-0.002	0.962	0.016	Factor3	-0.836
Α18	-0.847	-0.096	0.200	-0.026	Factor1	-0.839
Α19	-0.866	-0.088	0.178	-0.049	Factor1	-0.796
A1	0.102	0.007	-0.001	0.823	Factor4*	-0.811
A4	0.748	0.148	-0.217	0.033	Factor1	0.961
A6	0.805	0.176	-0.139	0.096	Factor1	0.962
A7	0.744	0.144	-0.162	0.030	Factor1	-0.847
A8	0.827	0.126	-0.180	0.056	Factor1	-0.866
A9	0.816	0.165	-0.141	0.110	Factor1	-0.814
A16	0.131	0.966	-0.012	-0.029	Factor2	-0.824
A17	0.107	0.970	-0.011	-0.014	Factor2	-0.771

Item Response Theory - Graded Response Model

Discrimination parameters (a) were high across most items. Within Factor 1, the strongest items were A19 (a = 4.05), A18 (a = 3.75), A8 (a = 3.55), A11 (a = 3.37), and A9 (a = 3.32). Thresholds (b1-b4) indicated that these items were most informative in the mid-to-high range of the latent trait (θ ≈ −2.0 to 0.4 for successive transitions). Factors 2 and 3 exhibited extremely high discrimination (a > 7 and a > 9, respectively), yet with localized information around the mean of θ. Factor 4 items showed moderate discrimination (a ≈ 3.1) with thresholds centered around −1.5 to 0.5 (Table 2).

**Table 2:** Item Response Theory (Graded Response Model) – Item Parameters Note. Items A4, A6, A7, A8, and A9 were reverse-coded prior to estimation. Reported values reflect scoring after recoding. Discrimination (a) and threshold parameters (b1–b4) are reported for each item, separately by subscale. Higher a- values indicate greater discrimination; thresholds (b1–b4) represent transition points across response categories. Factor 4 was excluded as separate subscale and retained only for the calculation of the total score (shown in light black).

Subscale	Item	a	b1	b2	b3	b4
Factor 1	Α3	2.999	-2.13	0.329	0.277	0.445
Factor 1	Α5	2.463	-2.341	0.301	0.281	0.527
Factor 1	Α10	3.306	-2.225	0.289	0.264	0.439
Factor 1	Α11	3.369	-2.17	0.375	0.203	0.407
Factor 1	Α12	2.843	-1.868	0.319	0.329	0.391
Factor 1	Α13	2.946	-2.026	0.313	0.337	0.413
Factor 1	Α18	3.747	-1.864	0.302	0.198	0.399
Factor 1	Α19	4.053	-2.002	0.273	0.249	0.4
Factor 1	A4	2.751	-1.914	0.429	0.203	0.436
Factor 1 (recoded)	A6	3.158	-2.501	0.319	0.373	0.41
Factor 1 (recoded)	A7	2.525	-2.115	0.404	0.309	0.44
Factor 1 (recoded)	A8	3.552	-1.935	0.358	0.273	0.376
Factor 1 (recoded)	A9	3.317	-2.162	0.366	0.251	0.411
Factor 2	A16	7.509	-0.359	0.278	0.274	0.289
Factor 2	A17	7.403	-0.316	0.285	0.278	0.28
Factor 3	Α14	9.411	-0.814	0.232	0.228	0.234
Factor 3	Α15	9.816	-0.629	0.228	0.208	0.22
Factor 4	A1	3.144	-1.467	0.433	0.273	0.459
Factor 4 (recoded)	Α2	3.056	-1.529	0.458	0.398	0.376

Reliability

Internal consistency coefficients are presented in Table 3. Reliability was excellent for Factor 1 (α = .963), Factor 2 (α = .955), and Factor 3 (α = .963), and moderate for Factor 4 (α = .529). Given its low internal consistency (α = .53) and minimal item pool (2 items), Factor 4 was not considered robust enough to be reported as a separate subscale (items A1-A2). Nevertheless, these items were conceptually aligned with the overall construct and demonstrated adequate item-total correlations. To preserve content breadth and avoid loss of information, they were retained when calculating the total section score.

The final structure therefore comprised three interpretable subscales: Ethical, Legislative and Personal Dimensions (Factor 1), Spiritual and Religious Influence (Factor 2), and Perceived Religious Acceptance (Factor 3), along with a reliable total section score (α = .905, 19 items; Table 3).

**Table 3:** Reliability Indices per Subscale and Total Score Note. Reliability was excellent for Factors 1-3 and moderate for Factor 4, as expected for a two-item scale. *Items A4, A6, A7, A8, and A9 displayed negative discrimination (a), indicating that their scoring direction was opposite to the underlying construct. To address this, these items, as well as Item A2, were reverse-coded. **Factor 4 was excluded as separate subscale and retained only for the calculation of the total score (shown in light black). The final questionnaire therefore contains three interpretable subscales (Ethical, Legislative and Personal Dimensions, Spiritual and Religious Influence and Perceived Religious Acceptance) alongside a reliable total score (total score α = .905, n=19 items).

Subscale	Items	Cronbach’s α
Factor 1 (Ethical, Legislative and Personal Dimensions)	Α3, A4*, Α5, A6*, A7*, A8*, A9*, Α10, Α11, Α12, Α13, Α18, Α19	0.963
Factor 2 (Spiritual and Religious Influence)	A16, A17	0.955
Factor 3 (Perceived Religious Acceptance)	Α14, Α15	0.963
Factor 4**	A1, Α2*	0.529
Total scale (n=19)	A1, Α2*, Α3, A4*, Α5, A6*, A7*, A8*, A9*, Α10, Α11, Α12, Α13, Α14, Α15, A16, A17, Α18, Α19	0.905

Test Information Functions (TIFs)

**Figure 1:**
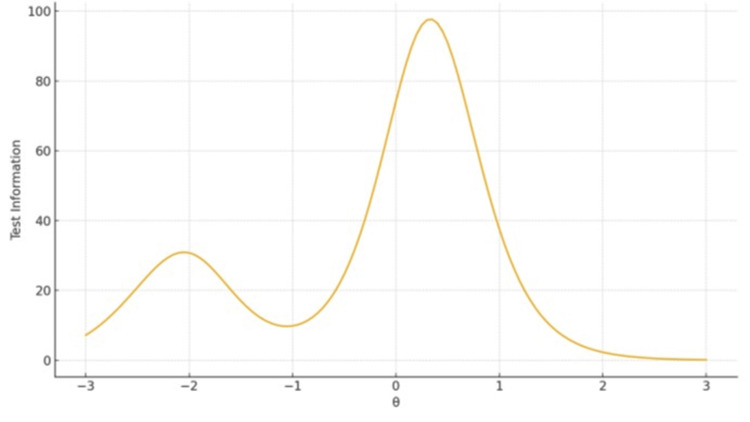
TIF plot for Factor 1 Ethical, Legislative and Personal Dimensions The x-axis represents the latent trait level (θ), ranging from −3 to +3 on the standard normal scale. The y-axis represents the Test Information value, reflecting measurement precision at each trait level.

**Figure 2:**
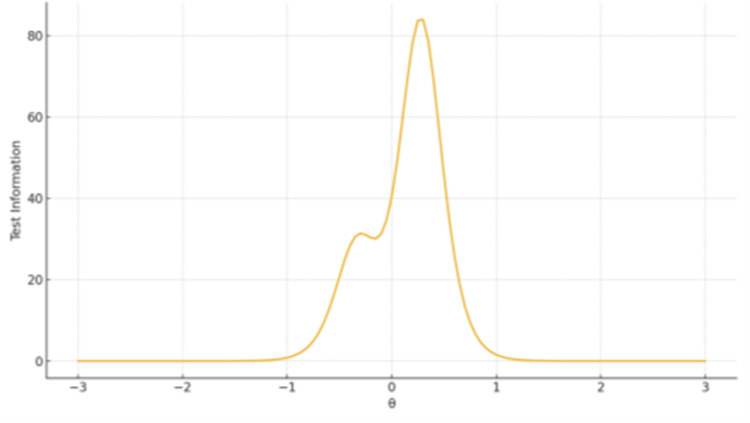
TIF plot for Factor 2 Spiritual and Religious Influence The x-axis represents the latent trait level (θ), ranging from −3 to +3 on the standard normal scale. The y-axis represents the Test Information value, reflecting measurement precision at each trait level.

**Figure 3:**
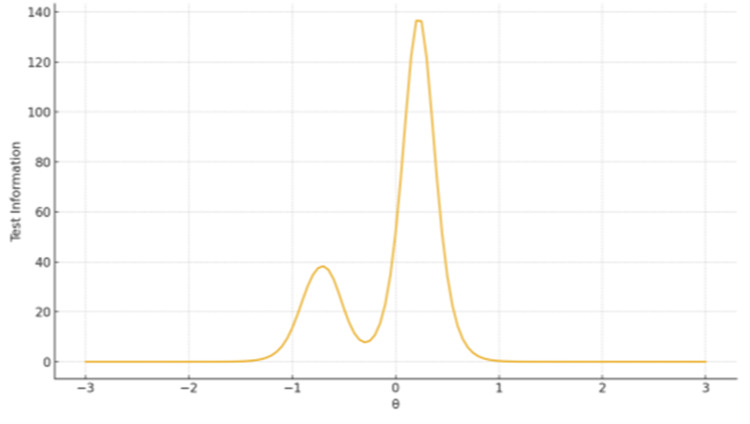
TIF plot for Factor 3 Perceived Religious Acceptance The x-axis represents the latent trait level (θ), ranging from −3 to +3 on the standard normal scale. The y-axis represents the Test Information value, reflecting measurement precision at each trait level.

TIFs confirmed broad coverage and high precision for Ethical, Legislative and Personal Dimensions (Factor 1), peaking around θ ≈ 0-1. The remaining two-item subscales displayed narrow information peaks - high sensitivity near the mean but limited coverage overall (Figures 1-3).

Perspectives on autonomous advance care planning

Exploratory Factor Analysis

A PCA with Varimax rotation was also conducted on 10 items of the section B of the questionnaire. The analysis supported a two-factor structure based on eigenvalues greater than 1. Items were assigned to factors according to their highest rotated loadings (Table 4). The rotated solution accounted for 63.4% of variance, revealing one dominant factor along with another one narrower subdimension.

**Table 4:** Exploratory Factor Analysis (PCA with Varimax) – Factor Loadings Note: Factor loadings ≥ 0.30 are shown in bold. The table indicates each item's primary factor (based on the highest loading) and the corresponding loading value. PCA = Principal Component Analysis

Item	Factor 1	Factor 2	Primary Factor	Loading
Β1	-0.726	-0.299	Factor1	0.810
Β2	-0.842	-0.243	Factor1	0.579
B3	0.579	-0.031	Factor1	-0.726
Β4	-0.812	-0.240	Factor1	-0.749
Β5	-0.555	-0.628	Factor2	0.529
Β6	-0.184	-0.844	Factor2	-0.842
Β7	-0.525	-0.655	Factor2	-0.812
Β8	-0.137	-0.841	Factor2	-0.628
Β9	-0.579	-0.065	Factor1	-0.844
Β10	-0.749	-0.349	Factor1	-0.655

Item Response Theory - Graded Response Model

Discrimination (a) and threshold parameters (b1-b4) for each item are reported in Table 5. Within Factor 1, the strongest items were B2 (a = 4.40), B4 (a = 3.56), and B10 (a = 3.16), all of which showed very high discrimination. Within Factor 2, items demonstrated consistently high discrimination, with B7 (a = 3.64), B6 (a = 3.49), and B5 (a = 3.24) performing particularly well. Threshold estimates indicated that most items provided the greatest information in the mid-to-high range of the latent trait (θ ≈ -2.0 to 0.5), reflecting the sensitivity of the questionnaire to variations around and slightly above the mean.

**Table 5:** Item Response Theory (Graded Response Model) – Item Parameters Note. Item B3 was reverse-coded prior to estimation. Reported values reflect scoring after recoding. Discrimination (a) and threshold parameters (b1–b4) are reported for each item, separately by subscale. Higher a -values indicate greater discrimination; thresholds (b1–b4) represent transition points across response categories.

Subscale	Item	a	b1	b2	b3	b4
Factor1 (recoded)	B3	1.422	-2.291	0.678	0.081	0.521
Factor1	Β1	3.001	-2.698	0.321	0.311	0.489
Factor1	Β10	3.161	-2.533	0.26	0.343	0.461
Factor1	Β2	4.402	-2.449	0.36	0.314	0.374
Factor1	Β4	3.559	-2.378	0.356	0.319	0.428
Factor1	Β9	1.319	-3.067	0.579	0.37	0.746
Factor2	Β5	3.241	-2.016	0.386	0.266	0.444
Factor2	Β6	3.493	-1.633	0.41	0.258	0.395
Factor2	Β7	3.643	-2.297	0.345	0.258	0.47
Factor2	Β8	2.78	-1.975	0.471	0.218	0.46

Reliability

Cronbach’s α coefficients indicated excellent reliability for the major subscale (α = .832). Reliability was moderate for the shorter subscale, which is expected due to the limited number of items. Detailed indices for each subscale are reported in Table 6. The final structure therefore comprises two interpretable subscales: Attitudes toward Advance Directives (Factor 1) and Healthcare Proxy Attitudes (Factor 2), alongside a reliable total section score (α = .883; 10 items; Table 6).

**Table 6:** Reliability Indices per Subscale and Total Score Note. *Item B3 displayed negative discrimination (a), indicating that its scoring direction was opposite to the underlying construct. To correct this, this item was reverse-coded. After recoding, all discrimination values became positive, and the items aligned psychometrically with the construct. The final questionnaire contains two interpretable subscales (Advance Directives and Health Care Proxy Attitudes) alongside a reliable total score (total score α = .883, n=10 items).

Subscale	Items	Cronbach’s α
Factor 1 (Advance Directives)	Β1, Β2, B3*, Β4, Β9, Β10	0.832
Factor 2 (Health Care Proxy Attitudes)	Β5, Β6, Β7, Β8	0.844
Total scale (n=10)	Β1, Β2, B3*, Β4, Β5, Β6, Β7, Β8, Β9, Β10	0.883

Test Information Functions

**Figure 4:**
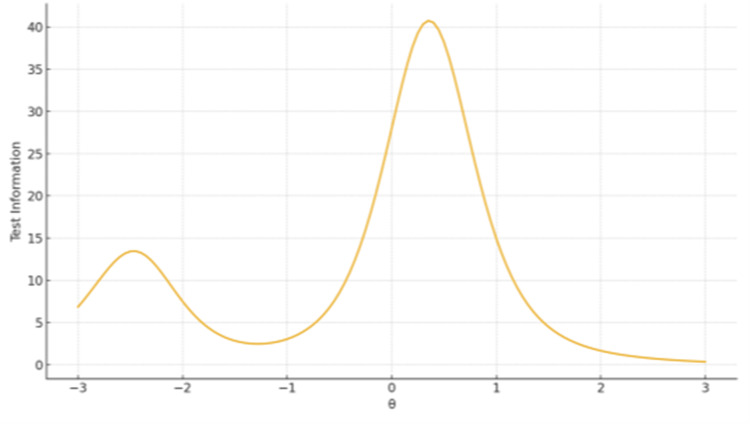
TIF plot for Factor 1 Advance Directives The x-axis represents the latent trait level (θ), ranging from −3 to +3 on the standard normal scale. The y-axis represents the Test Information value, reflecting measurement precision at each trait level.

**Figure 5:**
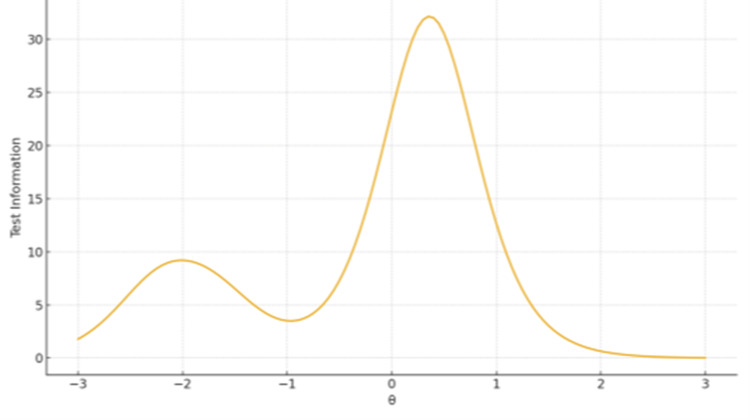
TIF plot for Factor 2 Health Care Proxy Attitudes The x-axis represents the latent trait level (θ), ranging from −3 to +3 on the standard normal scale. The y-axis represents the Test Information value, reflecting measurement precision at each trait level.

The main factor demonstrated broad information coverage across the θ continuum, with peak measurement precision around θ = 0 to 1. In contrast, the second subscale exhibited sharp but narrow information peaks, indicating high sensitivity near the average trait level (Figures 4-5).

Perspectives on end-of-life practices and palliative care

Exploratory Factor Analysis

PCA with Varimax rotation supported a five-factor solution (Table 7) for this section of the questionnaire. Items were allocated to factors based on their highest rotated loadings, with one dominant factor and four additional narrower ones. The rotated solution accounted for a substantial proportion of variance (62.0%).

**Table 7:** Exploratory Factor Analysis (PCA with Varimax) – Factor Loadings Note: Factor loadings ≥ 0.30 are shown in bold. The table indicates each item's primary factor (based on the highest loading) and the corresponding loading value. *Factor 4 (items C10–C11) showed inadequate internal consistency (α < .60) and was not retained as a separate subscale. These items are included only in the total section score. **Factor 5 (items C12–C14) showed inadequate internal consistency (α < .60) and was not retained as a separate subscale. These items are included only in the total section score. PCA = Principal Component Analysis α = Cronbach's alpha

Item	Factor 1	Factor 2	Factor 3	Factor 4*	Factor 5**	Primary Factor	Loading
C1	0.174	0.885	-0.053	-0.0	-0.019	Factor2	0.885
C2	0.200	0.905	-0.073	0.046	-0.005	Factor2	0.799
C3	0.192	0.868	-0.028	0.039	0.006	Factor2	0.629
C4	-0.018	0.785	-0.063	0.085	0.157	Factor2	-0.721
C5	0.635	0.241	-0.266	0.254	0.085	Factor1	-0.717
C6	0.73	0.225	-0.299	0.167	0.047	Factor1	-0.484
C8	0.641	0.216	-0.252	0.165	0.004	Factor1	-0.769
C10	-0.025	0.052	0.032	0.799	0.155	Factor4*	-0.741
C11	0.219	0.151	-0.099	0.629	-0.230	Factor4*	-0.348
C20	0.217	0.069	-0.916	-0.005	0.012	Factor3	-0.729
C21	0.216	0.072	-0.906	0.026	-0.011	Factor3	-0.407
C7	-0.385	0.398	-0.056	0.073	0.21	Factor2	0.905
C9	-0.685	-0.167	0.144	-0.009	-0.195	Factor1	-0.916
C12	-0.250	-0.038	0.01	0.087	-0.721	Factor5**	-0.906
C13	-0.324	-0.012	0.002	-0.139	-0.717	Factor5**	0.868
C14	-0.266	-0.122	-0.036	-0.115	-0.484	Factor5**	0.785
C15	-0.769	-0.122	0.155	0.045	-0.252	Factor1	0.635
C16	-0.741	-0.148	0.197	0.096	-0.225	Factor1	0.73
C17	-0.348	-0.244	-0.128	0.203	-0.08	Factor1	0.398
C18	-0.729	-0.174	0.18	0.015	-0.207	Factor1	0.641
C19	-0.407	-0.236	-0.328	-0.097	0.136	Factor1	-0.685

Item Response Theory - Graded Response Model

Discrimination parameters (a) were generally high, indicating effective differentiation along the latent trait, while threshold estimates (b1-b4) suggested that most items were most informative from the mid to higher ranges of θ. Within Factor 1, discrimination values varied considerably. The strongest items were C15 (a = 3.31), C18 (a = 3.18), and C6 (a = 3.27). Within Factor 2, most items demonstrated very strong discrimination. In particular, C2 (a = 6.35), C1 (a = 5.11), and C3 (a = 4.52) were excellent indicators, while C4 (a = 2.68) performed moderately well. Within Factor 3, both items (C20, a = 8.61; C21, a = 8.54) exhibited exceptionally high discrimination, providing extremely localized but powerful information. Within Factor 4, both items (C10, a = 2.64; C11, a = 2.60) showed moderate-to-strong discrimination, with thresholds positioned around θ ≈ -1.5 to 0.5, and within Factor 5, items C12 (a = 2.58) and C13 (a = 2.89) were the strongest contributors, while C14 (a = 1.89) was weaker but still acceptable.

Reliability

Cronbach’s α was excellent for the larger subscales and lower for shorter ones, as expected (see Table 8 for reliability indices and Table 9 for item parameters). Although the initial exploratory factor analysis of Section C yielded a five-factor solution, Factors 4 and 5, along with their corresponding items, were excluded as separate subscales due to their limited reliability and small item pools. Moreover, no total score was reported for Section C, as its overall internal consistency did not reach acceptable levels. The final Section C of the questionnaire, therefore, contains only three interpretable subscales: Personal and Normative Dimensions (Factor 1), Definitions and Moral Equivalence (Factor 2) and Religious Influence (Factor 3).

**Table 8:** Reliability Indices per Subscale *Items C5, C6, and C8 showed negative discrimination and were therefore reverse-coded. After recoding, all discrimination values became positive, and the items aligned psychometrically with the construct. Factors 4 and 5 were excluded as separate subscales due to poor performance (shown in light black). The final questionnaire therefore contains only three interpretable subscales (Personal and Normative Dimensions, Definitions and Moral Equivalence and Religious Influence).

Subscale	Items	Cronbach’s α
Factor 1 (Personal and Normative Dimensions)	C15, C16, C17, C18, C19, C5*, C6*, C8*, C9	0.856
Factor 2 (Definitions and Moral Equivalence)	C1, C2, C3, C4, C7	0.846
Factor 3 (Religious Influence)	C20, C21	0.951
Factor 4	C10, C11	0.316
Factor 5	C12, C13, C14	0.597

**Table 9:** Item Response Theory (Graded Response Model) – Item Parameters Note. Discrimination (a) and threshold parameters (b1–b4) are reported for each item, separately by subscale. Higher a -values indicate greater discrimination; thresholds represent transition points across response categories. Factors 4 and 5 were excluded as separate subscales (shown in light black).

Subscale	Item	a	b1	b2	b3	b4
Factor 1	C15	3.305	-2.647	0.29	0.388	0.517
Factor 1	C16	2.976	-2.535	0.265	0.4	0.489
Factor 1	C17	0.904	-4.621	0.591	0.321	0.878
Factor 1	C18	3.182	-2.876	0.342	0.298	0.499
Factor 1	C19	0.613	-3.869	0.905	1.234	0.422
Factor 1	C5	2.653	-3.462	0.525	0.476	0.505
Factor 1	C6	3.265	-2.554	0.376	0.36	0.44
Factor 1	C8	2.218	-2.412	0.515	0.298	0.503
Factor 1	C9	2.523	-2.885	0.254	0.378	0.58
Factor 2	C1	5.108	-1.193	0.329	0.257	0.37
Factor 2	C2	6.348	-1.096	0.299	0.261	0.314
Factor 2	C3	4.522	-1.159	0.339	0.238	0.392
Factor 2	C4	2.68	-1.632	0.477	0.362	0.53
Factor 2	C7	0.681	-5.334	1.238	0.797	1.308
Factor 3	C20	8.609	-0.611	0.236	0.233	0.232
Factor 3	C21	8.536	-0.737	0.245	0.227	0.257
Factor 4	C10	2.641	-1.273	0.542	0.349	0.38
Factor 4	C11	2.596	-1.469	0.553	0.415	0.445
Factor 5	C12	2.582	-2.769	0.362	0.327	0.577
Factor 5	C13	2.889	-2.556	0.382	0.364	0.534
Factor 5	C14	1.89	-1.937	0.47	0.266	0.622

Test Information Functions

**Figure 6:**
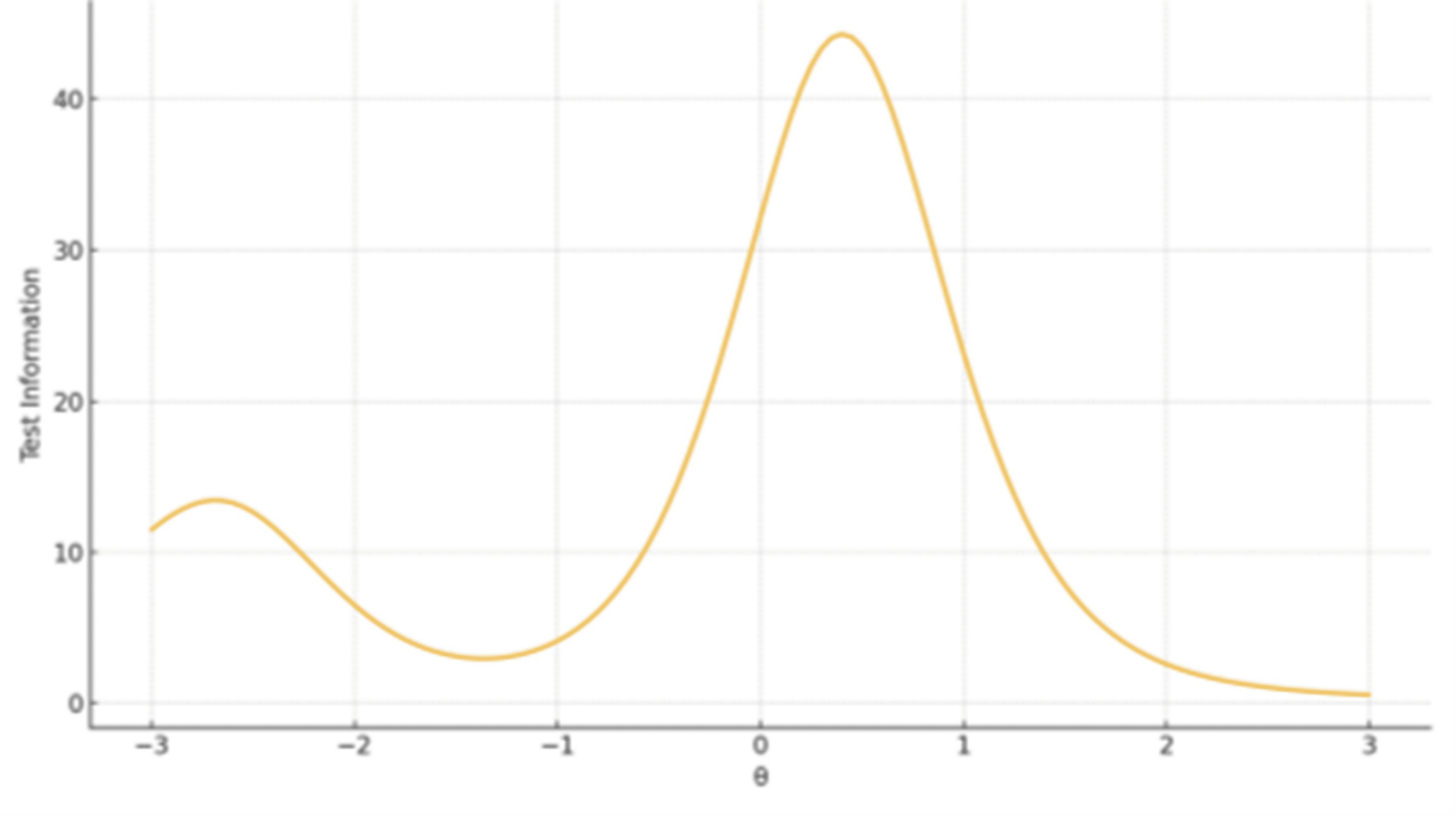
TIF plot for Factor 1 Personal and Normative Dimensions The x-axis represents the latent trait level (θ), ranging from −3 to +3 on the standard normal scale. The y-axis represents the Test Information value, reflecting measurement precision at each trait level.

**Figure 7:**
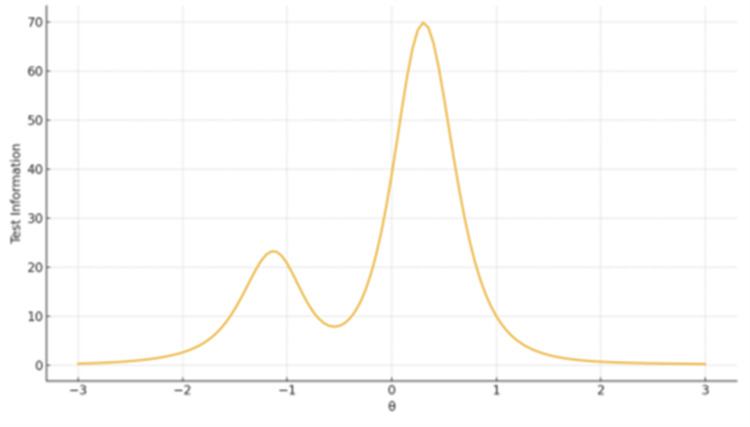
TIF plot for Factor 2 Definitions and Moral Equivalence The x-axis represents the latent trait level (θ), ranging from −3 to +3 on the standard normal scale. The y-axis represents the Test Information value, reflecting measurement precision at each trait level.

**Figure 8:**
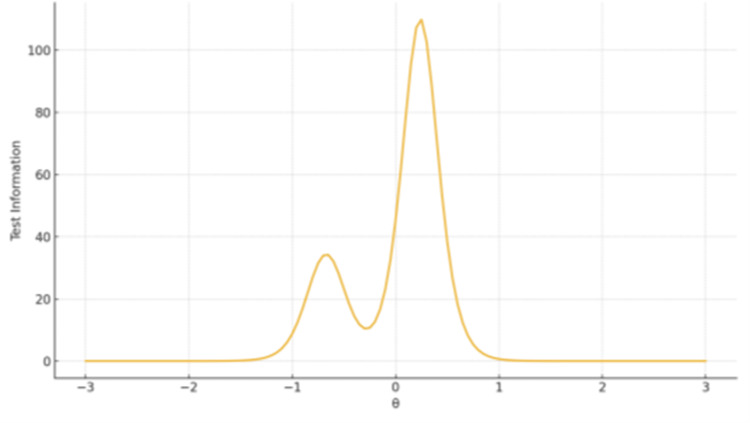
TIF plot for Factor 3 Religious Influence The x-axis represents the latent trait level (θ), ranging from −3 to +3 on the standard normal scale. The y-axis represents the Test Information value, reflecting measurement precision at each trait level.

TIF plots showed that the largest factor provides broad coverage with peak precision around θ ≈ 0-1, whereas shorter subscales produce narrow but sharp information peaks centered near the mean of θ, reflecting high sensitivity but limited breadth (Figures 6-8).

Existential Readiness and Death Acceptance Index (ERDAI)

In addition to its subscales, the PEACE-Q produces a total index score, the Existential Readiness and Death Acceptance Index (ERDAI). The ERDAI was calculated from the combined items of the three Sections (A, B, C) after excluding five items with low loadings from Section C, resulting in a final total of 45 items. This composite measure reflects the extent to which an individual demonstrates existential readiness - preparedness to confront and make informed decisions on EOL issues - and death acceptance.

Reliability for the ERDAI was excellent (α = 0.911; Table 10). High scores reflect individuals who acknowledge death as an inherent part of human existence and are willing to support autonomy and bioethical principles [50] through institutional or personal acts (e.g., drafting advance directives, appointing a healthcare proxy, endorsing palliative-only care, or consenting to the withdrawal of futile treatment). In contrast, lower scores may reflect greater dependence on external authorities (law, religion, family, clinicians) or difficulties to engage with EOL situations and the existential meaning of death.

**Table 10:** Descriptive Indices per Subscale and Total Index Score (ERDAI) Note: Values represent mean scores with standard deviations in parentheses. Higher scores indicate more favorable attitudes toward the respective dimension. M = Mean; SD = Standard Deviation; ERDAI = Existential Readiness and Death Acceptance Index

	Men n=349		Women n=697		Total N=1046	
	M	SD	M	SD	M	SD
Section A: Perspectives on Euthanasia and Physician-Assisted Dying						
Ethical, Legislative and Personal Dimensions	47.98	13.26	48.48	11.23	48.32	11.94
Spiritual and Religious Influence	3.54	1.75	3.79	1.76	3.71	176
Perceived Religious Acceptance	4.60	2.25	4.58	2.06	4.59	2.12
Total Section Score	61.82	13.93	62.36	11.65	62.19	12.46
Section B: Perspectives on Autonomous Advance Care Planning						
Attitudes toward Advance Directives	22.29	4.56	22.30	4.39	22.30	4.45
Healthcare Proxy Attitudes	14.15	3.64	13.50	3.49	13.72	3.55
Total Section Score	36.43	7.46	35.79	7.08	36.01	7.21
Section C: Perspectives on End-of-Life Practices and Palliative Care						
Personal and Normative Dimensions	33.83	5.99	33.95	5.54	33.91	5.69
Definitions and Moral Equivalence	14.32	4.62	14.12	3.89	14.19	4.14
Religious Influence	4.42	2.22	4.61	2.15	4.54	2.18
Existential Readiness and Death Acceptance Index (ERDAI)						
Total Index Score	150.84	23.16	150.85	19.95	150.84	21.06

Thus, the global score functions as an index of existential readiness, revealing not only what individuals believe but also how they incorporate death into the fabric of their personal narrative and sense of responsibility.

Descriptive Indices

Table 10 presents the descriptive indices (means and standard deviations) for men, women, and the total sample for each subscale and the ERDAI index of the PEACE-Q Scale.

Within Section A: Perspectives on Euthanasia and Physician-Assisted Dying, the subscales (Ethical, Legislative and Personal Dimensions, Spiritual and Religious Influence, and Perceived Religious Acceptance) yielded high mean scores across both genders. In Section B: Perspectives on Αutonomous Advance Care Planning, participants reported moderate scores on the relevant subscales (Attitudes toward Advance Directives and Healthcare Proxy Attitudes), whereas Section C: Perspectives on End-of-Life Practices and Palliative Care demonstrated mean scores consistent across men and women.

Finally, the Existential Readiness and Death Acceptance Index (ERDAI) produced a total score mean of 150.84 (SD = 21.06) across the sample, with virtually identical averages for men and women.

Test-retest Reliability

Three months later, the PEACE-Q was readministered to a randomly chosen subset of the original sample (N=210) to assess the test-retest reliability. At the retest period, Cronbach’s a was .882. The test-retest reliability was r = .88 (p<.001) for the total index ERDAI. On a subscale level, the correlation was: Perspectives on Euthanasia and Physician-Assisted Dying r = .87, Perspectives on Αutonomous Advance Care Planning r = .90, and Perspectives on End-of-Life Practices and Palliative Care r = .89.

## Discussion

The objective of this study was the development and initial psychometric validation of the PEACE-Q, designed as an internationally applicable instrument, with validation conducted in a large Greek population-based sample. The findings demonstrated it produces strong psychometric properties, including high internal consistency, robust factor structure and satisfactory test-retest reliability.

To our knowledge, this is the first validated instrument designed to capture public attitudes toward euthanasia, PAD, ADs and EOL care in a conceptually cohesive manner, reflecting broader societal perspectives beyond the clinician- or relative-based focus of earlier studies [34,51-57]. In contrast with existing instruments that focus on single dimensions or stakeholder groups [33-38], the PEACE-Q is designed to capture the interplay of societal attitudes encompassing personal, ethical, legislative, and spiritual dimensions. This multidimensional approach is crucial, as research consistently shows that attitudes toward euthanasia and PAD are influenced by a spectrum of factors, including religious beliefs, cultural background, and legal context [58-60]. For example, religiosity and personal beliefs are among the most significant predictors of attitudes toward euthanasia and PAD, often outweighing demographic variables such as age or education [58].

Importantly, the instrument's design enables adaptation to diverse legal and cultural frameworks - from jurisdictions where euthanasia and PAD remain illegal or underdeveloped to those with established regulatory systems. Its initial validation within the Greek context, characterized by strong religious traditions, underdeveloped legal framework and ongoing public debate about EOL issues, highlights its broader applicability and relevance not only in countries with established frameworks but also in culturally and systemically distinct contexts.

International evidence also highlights that even in countries where assisted dying is legal, cultural and religious diversity leads to a wide range of perspectives [60], underscoring the need for instruments that can capture this complexity and the full spectrum of relevant attitudes.

Psychometric evaluation through exploratory factor analysis (EFA) and item response theory (IRT) confirmed a multidimensional structure, with the questionnaire organized into three first-order sections, each comprising specific subscales that function as second-order factors. These sections can be meaningfully employed both independently and collectively. At the section level, researchers may focus on specific domains - for instance, attitudes toward legal regulation of euthanasia/PAD, willingness to prepare advance directives or perspectives toward the moral equivalence of EOL practices - depending on the research question and the sociocultural context addressed. These three dimensions and their corresponding subscales form the basis for calculating the global ERDAI, a composite score extending beyond isolated perceptions to reflect a holistic existential orientation toward end-of-life issues and death, encompassing the interplay of moral - personal perspectives and religious beliefs.

Taken together, the PEACE-Q provides a psychometrically robust and conceptually integrated instrument. Its multidimensional structure allows for nuanced exploration of ethical, legislative, and religious considerations across different societies and cultural contexts, while the ERDAI enables a holistic evaluation of existential readiness and acceptance of death at the population level internationally.

Limitations and further research

This study is not without limitations. As an initial validation study conducted in Greece, the use of the snowball sampling method may limit the generalizability of findings, as individuals more interested in bioethical debates may have been overrepresented. Moreover, the reliance on self-reported attitudes introduces the risk of social desirability bias, particularly in a cultural setting where EOL discussions are still taboo [26]. Finally, while test-retest reliability was satisfactory, longer-term stability remains to be evaluated.

Future studies should use CFA in independent samples to further validate the latent structure of the PEACE-Q. A critical next step should also involve cross-cultural validation of the PEACE-Q in diverse international settings - including jurisdictions with varying legal frameworks, different religious compositions and distinct healthcare systems.

Future research should also build upon these findings by employing longitudinal designs to examine stability and change of attitudes over time, assessing predictive validity by exploring whether PEACE-Q scores correspond to actual behaviors, such as applying the instrument in educational or policy interventions to determine its sensitivity in capturing attitude changes.

## Conclusions

In conclusion, the PEACE-Q emerges as a valid, reliable, and culturally sensitive instrument for assessing societal attitudes toward euthanasia, PAD, ADs, and EOL care. Its initial validation within the Greek setting demonstrates strong psychometric properties and establishes a foundation for international application. The instrument's flexible, multidimensional design enables adaptation to various settings while maintaining conceptual coherence and measurement integrity. Beyond its research utility, the PEACE-Q offers practical value for multiple stakeholders internationally, from jurisdictions where such issues remain underdeveloped to those with established regulatory systems. Findings derived from its application may inform clinical communication and policy development, guide the development of culturally sensitive training programs for healthcare professionals, foster evidence-based public debate and support the gradual integration or reform of relevant regulatory frameworks within healthcare systems across different countries. As a modular tool, it can address specific thematic sections according to local priorities, while the ERDAI composite score provides a comprehensive measure of existential orientation toward end-of-life issues at the population level. By bridging empirical research, bioethical deliberation, and policy-making, the PEACE-Q provides an essential tool for understanding and addressing end-of-life attitudes in diverse but interconnected contexts.

## Appendices

Full Version of the Physician-Assisted Dying, Euthanasia, Advance Directives and Care at the End-of-Life Questionnaire (PEACE-Q)

Section A: Perspectives on Euthanasia and Physician-assisted dying

Introduction

Euthanasia: As you may know, euthanasia is a legal procedure in several countries worldwide, and several hundred citizens undergo it every year.

Euthanasia is defined as the act of causing a painless death upon the explicit request of the individual. This person - often not hospitalized - usually suffers from an incurable disease with no prospect of improvement, making life unbearable. The request is submitted to a designated committee, which evaluates it and decides upon its approval or rejection.

The request can only be applied by the individual concerned, based on their own free will, provided they are fully conscious and mentally competent. Notable is the fact OR It is worth mentioning that no individual can request euthanasia on behalf of another person. Thus, such a request cannot be applied by doctors, relatives, friends or partners. In most cases several requests by the person concerned are required, separated by a time interval between them, along with comprehensive information provided to the applicant, regarding their choices, the consequences of their request and alternative solutions (e.g., palliative care).

Note 1: Euthanasia is usually performed by a doctor administering an injection to provide fast and painless death.

Physician-assisted dying (PAD): PAD shares many similarities with euthanasia, particularly in terms of the process leading to it. In PAD, however, the physician provides the patient with the means and instructions (e.g., lethal medication) to end their own life. The main difference from euthanasia is that death occurs through the patient's own action rather than through direct intervention by another person.

Note 2: In some countries, such as the Netherlands, Belgium and Luxembourg, both euthanasia and physician-assisted dying are legal procedures, subject to very strict conditions. In contrast, in Switzerland and some U.S. states, PAD is permitted but euthanasia remains illegal.

Typical circumstances under which individuals may request euthanasia or PAD include:

·       Advanced cancer with painful symptoms (cachexia, dyspnea, chronic pain)

·       Neurological diseases with bed rest (e.g., multiple sclerosis, severe Parkinson's disease, strokes, quadriplegia)

·       Conditions requiring permanent ventilator support at home (stroke, brain injury, severe respiratory failure, chronic neurological diseases, quadriplegia)

·       Patients with various debilitating diseases (severe heart failure, renal failure, cirrhosis, severe arthritis, Parkinson's disease - severe cachexia) causing inability to self-care and a painful daily life

·       Chronic, persistent and unbearable pain

·       Severe forms of arthritis (rheumatoid arthritis, degenerative osteoarthritis, etc.) with chronic persistent pain and joint destruction, causing inability to care for oneself

·       Inability to control bowel movements and urination

·       Severe chronic dyspnea with constant distress

·       Long-term institutionalization in nursing homes with inability for self-care

·       Absence of relatives, friends and companions

·       Blindness, deafness and inability to communicate

·       Quadriplegia (paralysis of the upper and lower limbs) after cervical injury or other severe spinal damage, resulting in permanent bed confinement

Please indicate your level of agreement by choosing the number (1, 2, 3, 4, 5) that best matches your opinion.

I STRONGLY DISAGREE

DISAGREE

NEITHER AGREE NOR DISAGREE

I AGREE

I STRONGLY AGREE

A1.  In my opinion, euthanasia and physician-assisted dying are the same.

1

2

3

4

5

A2.  For me, it is very important that, in physician-assisted dying, death results from an act by the individual rather than from an act by another person (a physician), as in euthanasia.

1

2

3

4

5

A3.  A physician should assist a terminally ill patient to terminate their life, provided that the patient requests it, their medical condition justifies it, and the law permits it.

1

2

3

4

5

A4.  In any case, a physician’s contribution in provoking death is contrary to the very nature of the medical profession.

1

2

3

4

5

A5.  In some medical conditions, terminating life could constitute a humane act of mercy and compassion.

1

2

3

4

5

A6.  Anyone who helps a terminally ill patient die is unjustifiable.

1

2

3

4

5

A7.    Terminating a person’s life, who suffers from serious illness but is not hospitalised, is unjustifiable in all cases, regardless of whether it was requested by the individual.

1

2

3

4

5

A8.  Physician-assisted dying is illegal in my country and is punishable by law. In my opinion, this is appropriate.

1

2

3

4

5

A9.         In my country, euthanasia is completely illegal and classified as “intentional homicide.” In my opinion, this is appropriate.

1

2

3

4

5

A10.        To what extent do you agree or disagree that legislation should permit euthanasia for individuals who meet specified criteria and conditions?

1

2

3

4

5

A11.        To what extent do you agree or disagree that legislation should permit physician-assisted dying for individuals who meet specified criteria?

1

2

3

4

5

A12.        I would seriously consider requesting physician-assisted dying if there were no other means of restoring my health and I was experiencing severe symptoms.

1

2

3

4

5

A13.        I would seriously consider requesting euthanasia if there were no other means of restoring my health and I was experiencing severe symptoms.

1

2

3

4

5

A14.        I believe that euthanasia is permitted by my religion.

1

2

3

4

5

A15.        I believe that physician-assisted dying is permitted by my religion.

1

2

3

4

5

A16.        Euthanasia would be an option for me only if permitted by my religion.

1

2

3

4

5

A17.        Physician-assisted dying would be an option for me only if permitted by my religion.

1

2

3

4

5

A18.        I would like to have the option to request physician-assisted dying if necessary.

1

2

3

4

5

A19.        I would like to have the option to request euthanasia if necessary.

1

2

3

4

5

Section B: Perspectives on Autonomous Advance Care Planning

Introduction

Advance directives (ADs): ADs are legally binding documents established in many countries (France, Germany, Spain, USA, South Korea, India, Australia, New Zealand). Through ADs, a mentally competent adult may formally record, on their own initiative, their wishes regarding future medical care and provide explicit instructions for key medical decisions, in case they later lose their competence. ADs are activated when the person is no longer able to express their wishes (e.g., coma) due to serious illness or injury. Their purpose is to guide physicians and relatives in the decision-making progress on the person’s behalf, thereby ensuring that such decisions remain consistent with the individual’s previously stated preferences.

Two main types exist, which can be combined:

1. Living wills: A legal (often notarised) document where individuals record in detail their wishes regarding medical care in case, they become incompetent (e.g., due to severe illness or injury). For example, a person may request that every possible treatment be pursued, even if futile, or, conversely, prohibit specific interventions, that do not offer the prospect of restoring health (e.g., ICU admission, intubation, mechanical ventilation, chemotherapy, transfusions, amputations). In the second case the aim is to prevent unnecessary suffering and pain from aggressive treatments, which would either not fully restore health or lead to unacceptable quality of life (e.g., permanent vegetative state or severe disability).

2. Healthcare proxies: A legal (often notarized) document through which a person designates another individual - usually a relative - to represent them and make decisions on their behalf if, due to illness or injury, they are unable to express their wishes.

Advance directives are usually signed:

A. By patients with serious chronic illnesses (severe heart failure with dyspnea, terminal cancer, rapidly progressing dementia, etc.). These patients usually prohibit specific intervention (e.g., ICU intubation), after the medical team monitoring their progress have informed them that such intervention will not improve their condition.

B. Healthy individuals wish to record their wishes in advance, in case a future serious illness or injury renders them incompetent (e.g., severe stroke, polytrauma). In such circumstances - if, for example, they are in coma and hospitalization cannot restore their health to a level they would deem acceptable - physicians and relatives can rely on advance directives to decide which treatments are permissible, ensuring that all decisions align with the person’s stated preferences.

Please indicate your level of agreement by choosing the number (1, 2, 3, 4, 5) that best matches your opinion.

I STRONGLY DISAGREE

DISAGREE

NEITHER AGREE NOR DISAGREE

I AGREE

I STRONGLY AGREE

B1.  How much do you agree or disagree that, in all cases, a patient's prior refusal to have their life prolonged indefinitely by technological means should be respected?

1

2

3

4

5

B2.   If I had the opportunity, while healthy, I would choose to prepare advance directives in case needed.

1

2

3

4

5

B3. Even if I had the opportunity to complete advance directives, I would still feel unprepared to do so.

1

2

3

4

5

B4. If I were diagnosed today with a very serious illness, I would choose to prepare advance directives.

1

2

3

4

5

B5. I would agree to be appointed as a healthcare proxy by a loved one only if they had already prepared a living will reflecting their wishes or were willing to do so in the future.

1

2

3

4

5

B6. I would agree to be appointed as a health care proxy by a loved one, even if they were not willing to prepare a corresponding living will.

1

2

3

4

5

B7. If I were to prepare a living will, I would also appoint someone close to me as my proxy to ensure its implementation and to represent me in important decisions.

1

2

3

4

5

B8. I would appoint someone close to me - if I had one - as my health care proxy, even without preparing a living will that records my wishes.

1

2

3

4

5

B9. I would appoint someone close to me - if I had one - as my health care proxy only in conjunction with a living will that specifies my wishes.

1

2

3

4

5

B10. To what extent do you agree or disagree that legislation should allow the preparation of living wills and health care proxies for anyone who wishes to do so?

1

2

3

4

5

Section C: Perspectives on End-of-Life Practices and Palliative Care.

Introduction

End-of-life practices and relevant decisions aim to avoid unnecessary, futile and painful suffering caused by aggressive medical interventions disproportionate to the expected benefit. They are applied to patients with severe illness, unresponsive to treatment, with no prospect of recovery or survival. These practices are legally recognized in most European countries (e.g., France, Germany, the Netherlands, Belgium, the UK), as well as in the United States and Australia, and include:

A. Withholding of life-sustaining treatment: Decision not to initiate additional treatments (e.g., dialysis, intubation, mechanical ventilation, ICU admission) or not to intensify existing ones when deemed medically non-beneficial. Such a decision is based on scientific knowledge and clinical data, according to which these treatments are considered futile and ineffective for the patient's survival and recovery.

B. Withdrawal of life-sustaining treatment: Decision to discontinue all or part of the treatment administered, without replacing it with an equivalent alternative, as it is considered futile and ineffective for the patient's survival and recovery.

C. Do-not-resuscitate (DNR) order: Instruction recorded in the patient's medical file, to not attempt cardiopulmonary resuscitation (CPR) in case of cardiac arrest, recognising that most patients who survive in-hospital CPR remain in a vegetative state, with very few returning to their previous health levels.

Decisions on the application of the above practices (A, B and C) are made jointly by patients and physicians, or, if the patient is incapacitated, by their relatives together with physicians. The latter explains to the patient or, if the patient is comatose, to their family that, according to medical evidence, there is no prospect of survival or, in case of survival, the patient would remain with severe psychomotor disabilities.  Afterwards they recommend, subject to the patient’s or family’s agreement, either to issue DNR order or to withhold or withdraw life-sustaining treatment that cannot restore health or alter the trajectory toward death. At this stage, existing directives (living wills and/or health proxies) explored in the previous Section are very important as they clearly and safely guide physicians’ and relatives’ decisions, relieving them of uncertainty and speculation upon the patient's wishes.

Note 1: These practices do not aim to cause death (as in euthanasia). On the contrary, their purpose is to minimise suffering caused by futile interventions. Should death occur, it would be from the underlying disease, not from intentional intervention.

Palliative care mainly includes 1) medical management of pain, dyspnea, and other distressing symptoms in incurable disease, 2) psychological support for the patient and their relatives by psychologists and social workers, and 3) spiritual counselling and support aligned with the patient’s religious beliefs. Its aim is to alleviate suffering and reduce the psychological and physical distress they experience due to their incurable illness, improving and maintaining their quality of life.

Palliative care may be provided:

A.  When no curative options remain, even if life-sustaining treatments continue (e.g., mechanical ventilation, ICU care).

B. When the patient, due to their condition, is not conscious enough to express their wishes and has prohibited the application of specific treatment through prior directives (such as ICU admission, intubation and mechanical ventilation, artificial hydration and feeding, dialysis, etc.).

C. When a competent patient refuses a specific treatment.

D. After having taken the decision to apply end-of-life practices (withholding or withdrawal of treatment or  DNR order).

Note 2: Palliative care may be the next step following the decision to apply one of the above practices. For example, a patient may decide not to receive further treatment (withholding treatment) if it is deemed futile and to proceed exclusively with palliative care. 

Note 3: The term “terminal stage” refers to advanced incurable illness, with short expected survival.

Please indicate your level of agreement by choosing the number (1, 2, 3, 4, 5) that best matches your opinion.

I STRONGLY DISAGREE

DISAGREE

NEITHER AGREE NOR DISAGREE

I AGREE

I STRONGLY AGREE

C1.  In my view, withdrawing treatment is essentially equivalent to euthanasia.

1

2

3

4

5

C2.  In my view, withholding treatment is essentially equivalent to euthanasia.

1

2

3

4

5

C3.  In my view, a do-not-resuscitate (DNR) order is essentially equivalent to euthanasia.

1

2

3

4

5

C4.  The three end-of-life practices are, essentially, the same.

1

2

3

4

5

C5.  End-of-life practices should be prohibited.

1

2

3

4

5

C6.  End-of-life practices are contrary to my moral values.

1

2

3

4

5

C7.  The three end-of-life practices mentioned above are morally equivalent.

1

2

3

4

5

C8.  I have reservations about whether it is ethical to withdraw treatment from a patient in the final stages of illness.

1

2

3

4

5

C9.  A terminally ill patient should have the right to refuse treatment that merely prolongs life.

1

2

3

4

5

C10. Decisions regarding the application of end-of-life practices to a terminally ill patient who is unable to express their wishes should be made exclusively by physicians.

1

2

3

4

5

C11. Decisions regarding the application of end-of-life practices to a terminally ill patient who is unable to express their wishes should be made exclusively by the patient’s family.

1

2

3

4

5

C12. Decisions regarding the application of end-of-life practices to a terminally ill patient who is unable to express their wishes should be made jointly by the patient’s family and physicians.

1

2

3

4

5

C13. If a loved one were terminally ill and unable to express their wishes, I would want to be involved in the decision-making process.

1

2

3

4

5

C14. Nursing staff should be involved in decision-making processes regarding end-of-life practices.

1

2

3

4

5

C15. If a loved one were in the final stages of a serious illness, I would support their decision to use any of the end-of-life practices described above.

1

2

3

4

5

C16. I would request the application of one or more end-of-life practices if I were in a terminal condition with severe symptoms.

1

2

3

4

5

C17. To what extent do you agree or disagree that only palliative care should be provided when the progression toward death in a terminally ill patient cannot be reversed?

1

2

3

4

5

C18. A legal framework should exist to legitimise end-of-life practices and regulate the conditions for their implementation.

1

2

3

4

5

C19. In my opinion, end-of-life practices as described above are permitted by my religion.

1

2

3

4

5

C20. My religion would play an important role if  should I consider to request  an end-of-life practice for myself.

1

2

3

4

5

C21. My religion would play an important role if  should I decide to implement an end-of-life practice for a relative.

1

2

3

4

5
